# The LIPPSMAck POP (Lung Infection Prevention Post Surgery - Major Abdominal - with Pre-Operative Physiotherapy) trial: study protocol for a multi-centre randomised controlled trial

**DOI:** 10.1186/s13063-015-1090-6

**Published:** 2015-12-15

**Authors:** Ianthe Boden, Laura Browning, Elizabeth H. Skinner, Julie Reeve, Doa El-Ansary, Iain K. Robertson, Linda Denehy

**Affiliations:** Physiotherapy Department, Launceston General Hospital, Charles St, Launceston, Tasmania 7250 Australia; Department of Physiotherapy, Melbourne School of Health Sciences, University of Melbourne, Parkville, Victoria 3010 Australia; Division of Allied Health, Western Health, Furlong Road, St Albans, Victoria 3021 Australia; Department of Physiotherapy, Western Health, 160 Gordon St, Footscray, Victoria 3011 Australia; Department of Physiotherapy, School of Clinical Sciences, Faculty of Health and Environmental Sciences, Auckland University of Technology, Private Bag 92006, Auckland, 1142 New Zealand; Physiotherapy Department, North Shore Hospital, Waitemata District Health Board, North Shore City, Auckland 0622 New Zealand; School of Health Sciences, University of Tasmania, Locked Bag 1320, Launceston, Tasmania 7250 Australia; Clifford Craig Medical Research Trust, Launceston General Hospital, Charles Street, Launceston, Tasmania 7250 Australia

**Keywords:** Physiotherapy, Abdominal surgery, Breathing exercises, Post-operative pulmonary complication, Pre-operative education, Prevention, Randomised controlled trial

## Abstract

**Background:**

Post-operative pulmonary complications are a significant problem following open upper abdominal surgery. Preliminary evidence suggests that a single pre-operative physiotherapy education and preparatory lung expansion training session alone may prevent respiratory complications more effectively than supervised post-operative breathing and coughing exercises. However, the evidence is inconclusive due to methodological limitations. No well-designed, adequately powered, randomised controlled trial has investigated the effect of pre-operative education and training on post-operative respiratory complications, hospital length of stay, and health-related quality of life following upper abdominal surgery.

**Methods/design:**

The Lung Infection Prevention Post Surgery - Major Abdominal- with Pre-Operative Physiotherapy (LIPPSMAck POP) trial is a pragmatic, investigator-initiated, bi-national, multi-centre, patient- and assessor-blinded, parallel group, randomised controlled trial, powered for superiority. Four hundred and forty-one patients scheduled for elective open upper abdominal surgery at two Australian and one New Zealand hospital will be randomised using concealed allocation to receive either i) an information booklet or ii) an information booklet, plus one additional pre-operative physiotherapy education and training session. The primary outcome is respiratory complication incidence using standardised diagnostic criteria. Secondary outcomes include hospital length of stay and costs, pneumonia diagnosis, intensive care unit readmission and length of stay, days/h to mobilise >1 min and >10 min, and, at 6 weeks post-surgery, patient reported complications, health-related quality of life, and physical capacity.

**Discussion:**

The LIPPSMAck POP trial is a multi-centre randomised controlled trial powered and designed to investigate whether a single pre-operative physiotherapy session prevents post-operative respiratory complications. This trial standardises post-operative assisted ambulation and physiotherapy, measures many known confounders, and includes a post-discharge follow-up of complication rates, functional capacity, and health-related quality of life. This trial is currently recruiting.

**Trial registration:**

Australian New Zealand Clinical Trials Registry number: ACTRN12613000664741, 19 June 2013.

## Background

Elective upper abdominal surgery (UAS) is planned surgery involving an open incision above or extending above the umbilicus [1] and is predominately performed to remove cancerous tissue. Approximately 500 to 1000 procedures per 100,000 head of population are performed annually in developed countries [2, 3]. The most common complication following UAS is a post-operative pulmonary complication (PPC) [4] with a reported incidence of 13–53 % [5–10]. This is higher than the incidence for other major surgical procedures such as open lung resection, cardiac surgery via sternotomy, open lower abdominal surgery, and orthopaedic surgery [11–13]. A PPC is either a specific respiratory complication such as pneumonia or an undefined respiratory dysfunction that is clinically significant, compromises a patient’s predicted recovery, and requires additional medical management [14]. The variability in PPC rates following UAS may be explained by the differing studied patient risk profiles and PPC definitions utilised.

Respiratory pathophysiological changes after UAS are well reported, including atelectasis, impaired mucociliary clearance, diaphragm dysfunction, reduced lung volumes, and respiratory muscle and cough strength deficiencies [15–28]. These can contribute to bacterial proliferation and/or severe atelectasis [17, 29], thus increasing respiratory infection risk [14, 15]. PPCs are associated with increased morbidity, mortality, hospital expenditure, and length of stay (LOS) [5, 30–32]. Strategies to prevent PPCs should remain a high priority [33] due to their relatively high prevalence, relationship to poor patient outcomes, and increased health care costs.

Preventative non-pharmaceutical therapies such as coached deep breathing and coughing (DB&C) exercises and early ambulation are traditionally provided to patients following UAS [34]. Additionally, incentive spirometers [35], positive expiratory pressure (PEP) devices [36], and non-invasive ventilation (NIV) [37] can be utilised. These are often delivered by physiotherapists [8, 38], though in countries where physiotherapists are not involved with this patient group, this type of respiratory therapy is provided by nurses, doctors, or other health professionals [36, 39]. However, the efficacy of post-operative respiratory therapy to prevent PPCs following UAS is controversial. Systematic reviews and meta-analyses have concluded that lung expansion exercises including DB&C [40], incentive spirometry [35], and PEP [41] are of little benefit in reducing PPCs, with only NIV considered efficacious [37, 42]. Specifically, when post-operative ambulation is standardised, the addition of DB&C exercises does not reduce the incidence of PPCs in addition to assisted early ambulation alone [7, 10]. However, almost all clinical trials have included pre-operative physiotherapy (Pre-Op) education and training as usual care delivery to all participants. It is possible that this intervention alone may have independently reduced the risk of a PPC.

Evidence from six clinical trials [43–48] suggests that a single Pre-Op education session may reduce PPC rates by up to 78 % [47, 48] after UAS. However, these trials have methodological limitations, including small sample sizes, inconsistent end points, generalisability restrictions (single-centre trials, predominantly low-risk patient groups), sources of bias (non-random sampling, unblinded assessors and Hawthorne effects), and non-standardisation or reporting of potential confounders. These methodological limitations bring the reported effect on PPC rates with pre-operative physiotherapy education into question.

Even if the reported benefit on PPC rates is a true effect, it is not known if Pre-Op education and training would be effective in the context of recent advances in perioperative management such as Enhanced Recovery After Surgery (ERAS) guidelines. This multimodal package of 10–18 care elements provides significant improvements in complication rates and LOS [49]. Pre-operative education and lung expansion training are strongly recommended within ERAS guidelines, although it is acknowledged that evidence to support this specific element is weak [50]. Additionally, Pre-Op physiotherapy interventions previously studied were predominantly provided the day before surgery. This may not reflect current practice where, in many centres, patients attend a multi-disciplinary assessment clinic one to 6 weeks before their operation [51–53]. It is unknown whether Pre-Op physiotherapy education provided at these longer time intervals might also produce the previously reported effect on PPC prophylaxis.

Surveys of physiotherapy services to UAS patients in Australia have shown a stark reduction in hospitals (20 % down to 5 %) providing Pre-Op physiotherapy over the past 15 years [34, 54]. The reasons for this disinvestment of services are unknown. There are no cost-benefit analysis studies investigating physiotherapy to reduce respiratory complications, so conclusive evidence to inform the allocation of physiotherapy services to pre-operative education and training is lacking. Additionally, only short-term outcomes have been assessed. Reducing PPCs during the acute hospital stay may also improve important patient-focused longer term outcomes such as health-related quality of life (HRQoL) and physical capacity following discharge.

Considering the relative high incidence of PPCs following major UAS and the benefit to both the patient and the health care system if these were reduced, a well-designed, adequately powered trial is needed to determine both the clinical effect and cost benefit that Pre-Op physiotherapy education and training may, or may not, have on reducing PPC incidence following major UAS. Results will guide future cost-effective allocation of services to patients who require UAS.

### Trial objectives

The primary objective of the Lung Infection Prevention Post Surgery - Major Abdominal - with Pre-Operative Physiotherapy (LIPPSMAck POP) trial is to estimate the effect that Pre-Op physiotherapy education and training has on the incidence of PPCs following major UAS, when compared to an information booklet alone. Secondary objectives are to evaluate the effect of Pre-Op physiotherapy on hospital and ICU LOS, hospital costs, incidence of pneumonia, unplanned ICU admissions, time to early ambulation, readiness to discharge from hospital, and, at six weeks following surgery, patient-reported complications, HRQoL, and functional capacity.

## Methods/design

### Trial design

**Fig. 1:**
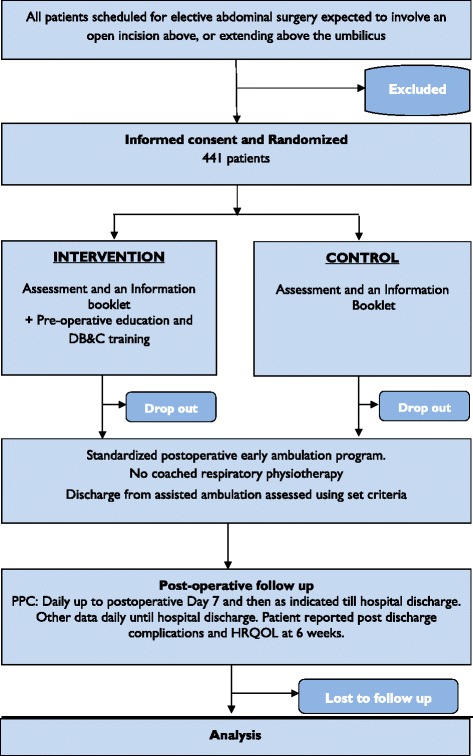
CONSORT flow diagram of the LIPPSMAck POP clinical trial. DB&C deep breathing and coughing, PPC post-operative pulmonary complications, HRQOL health-related quality of life

The LIPPSMAck POP trial is a pragmatic, investigator-initiated, bi-national, multi-centre, randomised controlled, parallel group, clinical trial. It is patient- and assessor-blinded, and powered for superiority. Eligible patients will be randomly assigned via concealed allocation to receive 1) a pre-operative assessment by a physiotherapist and provision of an information booklet (control) or 2) a pre-operative assessment, information booklet, plus an additional education and DB&C training session by a physiotherapist (intervention). Post-operative respiratory physiotherapy and assisted early mobilisation will be standardised for both groups. See Fig. 1 for a CONSORT diagram of the LIPPSMAck POP trial and Table 1 for an overview of the trial methods and design.

**Table 1:** World Health Organisation (WHO) Trial Registration Data Set for LIPPSMAck POP trial

Data category	Information
Primary registry and trial identifying number	Australian New Zealand Clinical Trials Registry number: ACTRN12613000664741
Date of registration in primary registry	19/6/2013
Secondary identifying numbers	n/a
Trial protocol version	This is Version 4 of the protocol and was enacted on June 2013
Source(s) of monetary or material support	Clifford Craig Medical Research Trust ($60,000 AUD)
	University of Tasmania, virtual Tasmanian Academic Health Precinct ($50,000 AUD)
	Waitemata District Health Board and Three Harbours Health Foundation ($20,000 NZD)
	Tasmanian Health Service - Northern Region ($120,000 AUD)
Primary sponsor	Tasmanian Health Service - Northern Region
Secondary sponsor	Waitemata District Health Board
Contact for public queries	IB, ianthe.boden@ths.tas.gov.au
Contact for scientific queries	IB, ianthe.boden@ths.tas.gov.au
Public title	Pre-operative physiotherapy education for the prevention of chest infections following major abdominal surgery
Scientific title	LIPPSMAck POP trial – Pre-operative physiotherapy education for the prevention of post-operative pulmonary complications following major upper abdominal surgery: a bi-national, multi-centre, randomised, double-blinded placebo controlled trial.
Countries of recruitment	Australia, New Zealand
Health condition(s) or problem(s) studied	Pulmonary complications following major upper abdominal surgery
Intervention(s)	Active comparator: Pre-operative physiotherapy education and training
	Placebo comparator: Education booklet
Key inclusion and exclusion criteria	Ages eligible for study: ≥ 18 years
	Sexes eligible for study: both
	Accepts health volunteers: No
	Inclusion criteria: All adults awaiting elective upper abdominal surgery involving an open incision above the umbilicus.
	Exclusion criteria: 1. Any pre-existing condition that would limit ability to participate in the standardised post-operative mobilisation protocol. Defined as any person unable to stand upright and walk for a maximum of 1 min without a seated rest. 2. Unable to understand verbal instructions in English. 3. Unable to attend a pre-admission assessment and education session with a physiotherapist. 4. Open abdominal hernia repairs.
Study type	Type: Investigator initiated, interventional, non-pharmacological, pragmatic, study
	Allocation: Concealed randomisation
	Intervention model: parallel assignment
	Masking: patient and assessor blinded
	Primary purpose: Prevention
	Phase: Phase III
Date of first enrolment	24/6/2013
Target sample size	441
Recruitment status	Recruiting
Primary outcome(s)	Post-operative pulmonary complications during the first 14 days of the hospital stay
Key secondary outcomes	Pneumonia, length of hospital stay, hospital costs, day of ambulation >10mins, length of ICU stay, ICU readmission, post-operative adverse events, day to discharge from post-operative physiotherapy services, patient-reported complications, health-related quality of life, and physical capacity at 6 weeks following discharge from hospital.

### Trial setting

The three participating centres: the Launceston General Hospital (Launceston, Tasmania, Australia), North Shore Hospital (Auckland, New Zealand), and North West Regional Hospital (Burnie, Tasmania, Australia), represent a range of public hospital types. The North West Regional Hospital is a 240-bed rural secondary referral hospital; the Launceston General Hospital is a 330-bed inner-regional, primary referral hospital; and the North Shore Hospital is a 600-bed metropolitan, primary referral hospital. North Shore Hospital has also implemented Enhanced Recovery After Surgery (ERAS) guidelines to all surgical units. All hospitals are government funded, university affiliated, teaching hospitals.

Patients undergoing elective UAS at the participating centres attend an outpatient Pre-Admission Clinic (PAC) session one to six weeks prior to their operation where they are assessed by a multi-disciplinary team consisting of, as a minimum, a registered nurse, anaesthetist, and doctor from the admitting surgical team. Information about the surgical process, pain management, post-operative drips and drains, and expected recovery process are provided as standard care. Whereas, Pre-Op physiotherapy education and training at PAC is not normally provided, post-operative respiratory therapy and assisted ambulation by a physiotherapist are provided as standard care at the participating centres.

Each participating hospital’s institutional review board has approved the trial (the Human Research Ethics Committee (Tasmania) Network, Tasmania, Australia (protocol reference: H0011911), the Health and Disability Ethics Committee, New Zealand (protocol reference: 14/NTA/233)). The trial is conducted in accordance with the Declaration of Helsinki and was prospectively registered on 19 June 2013 at the Australian New Zealand Clinical Trials Registry (http://www.anzctr.org.au): ACTRN12613000664741.

### Eligibility and exclusion criteria

Eligible participants are patients over the age of 18 years attending PAC at the participating centres who are scheduled for UAS expecting to require an abdominal incision longer than 5 cm that will be above, or extending above, the umbilicus (Table 2) and requiring a minimum overnight hospital stay.

**Table 2:** List of eligible upper abdominal surgical procedures

Surgical category	Procedure
Colorectal	Anterior resection
	AP resection
	Hartmanns
	Hemicolectomy
	Low anterior resection
	Laparoscopic (+/−hand) assisted colectomy
	Partial colectomy
	Proctocolectomy
	Reversal of Hartmanns
	Sigmoid colectomy
	Small bowel resection
	Subtotal colectomy
	Total colectomy
Upper gastrointestinal	Gastrectomy
	Liver resection
	Oesophagectomy
	Open cholecystectomy
	Open hiatus hernia repair
	Pancreatic surgery
	Whipples
Urology	Adrenalectomy
	Cystic duct excision
	Nephrectomy
	Laparoscopic +/− hand assisted nephrectomy
	Pyeloplasty
	Radical cystectomy +/− ileal conduit
	Radical cystoprostatectomy
Other	Explorative laparotomy
	Splenectomy

Patients are excluded for any of the following criteria: (i) unable to understand verbal instructions in English; (ii) unable to participate in a single pre-admission session with a physiotherapist; (iii) requiring emergency surgery; (iv) a current hospital patient for a separate episode of care; (v) requiring organ transplant; (vi) open abdominal hernia repairs (hernia repairs are generally low-risk procedures which frequently do not involve extensive visceral manipulation and have fewer complications [55]); (vii) being unable to stand upright and ambulate for a maximum of 1 min.

### Randomisation and allocation

An administration assistant independent to the trial will prepare 441 sequentially numbered (1 to 441) opaque envelopes each containing an allocation card wrapped in extra paper or aluminium foil [56]. Allocation sequence is determined by a web-based computer generated (http://www.randomizer.org/) blocked random number table (7 blocks of 63; 1 = intervention, 2 = control). The randomisation tables are then sealed in an opaque envelope, locked within the research institute, and made unavailable to trial personnel. The number of consecutively numbered envelopes provided to each site will be dependent on funding agreements (that is, funded to recruit one block of 63 participants or, on a per patient recruit basis, until the end of the trial).

Local investigators will screen elective surgery and PAC lists daily for eligible patients who will be met face to face by local investigators at their PAC appointment. Informed consent will be obtained from potential participants; each eligible participant will be provided with a trial information sheet which is explained verbally to them and will be invited to participate. Those agreeing will sign a consent form as required by local ethics committees and in accordance with the Declaration of Helsinki. Where the local investigator or eligible patient is unable to attend PAC, the latter will be contacted by telephone and invited to enter the trial. The information and consent form will be mailed by post for signing, and the participant will be requested to bring them to hospital on the day of their operation.

Once informed consent has been obtained and the consent form signed, the pre-operative physiotherapist receives the group allocation for participants by opening the next sequentially numbered sealed opaque envelope containing the randomised group allocation. Patient details will be written on the envelope once opened to ensure that patients are randomised in the same order as recruited and the envelopes filed securely along with the consent form. Potential selection bias will be studied by extracting basic demographic data and planned surgical procedure from all excluded patients’ medical records.

### Trial interventions

Consenting participants will be randomly assigned to receive either i) a pre-operative assessment from a physiotherapist and provision of an information booklet (control) or ii) an additional education and DB&C training session (intervention).

### Control group

Participants will have a standardised assessment conducted by a physiotherapist consisting of: questioning on current health co-morbidities, mobility and functional status, smoking history, lung auscultation, subjective assessment of cough quality and strength, sputum production and colour, hand grip strength, Rapid Assessment of Physical Activity (RAPA) [57] and Specific Activity Questionnaire (SAQ) [58] to determine current activity and fitness levels, and Short Form 36 (SF-36 V2) [59] to measure HRQoL (see Data Collection section for further details). Participants will then be provided with an education booklet. This colour booklet contains written and pictorial information about abdominal surgery, expected types of pain management, medical lines and drains, post-operative recovery process, and how to prevent post-operative respiratory complications with early ambulation and self-directed DB&C exercises. The booklet includes detailed written instructions to perform DB&C exercises for two sets of 10 deep breaths followed by three coughs every hour during waking hours. Participants will be instructed to bring the booklet to hospital for reference following the operation. The contents of the booklet will not be discussed with participants in the control group and there will be no additional physiotherapy provided pre-operatively.

### Intervention group

Intervention group participants will be assessed and provided with an information booklet as per the control group and will then receive an additional single education and training session of approximately 30 min with a physiotherapist. Participants will be given an estimate of their likelihood of a PPC based on a risk prediction tool [5] and educated about the effect of anaesthesia, UAS, and bed rest on mucociliary clearance and lung volumes [19, 20]. To ameliorate these factors and prevent bacteria stagnation [15, 16] the importance of participating in an early post-operative ambulation program and performing self-directed DB&C exercises will be emphasised. Participants will be informed that a physiotherapist will assist them to walk as soon as possible on the first post-operative day, aiming for a duration longer than 10 min and at a pace causing mild breathlessness. Outside these assisted sessions, participants will be advised to walk or exercise by their bedside as frequently as they are able.

As it is frequently not possible to ambulate as early and as often as recommended to assist in preventing respiratory complications [8, 60], participants will be educated on the necessity of performing self-directed breathing exercises to protect their lungs following their operation. They will be instructed to perform DB&C exercises immediately from waking from the anaesthetic and then every hour during daytime waking hours until their first ambulation session, and then at any time when they are not ambulant. The physiotherapist will coach each participant in at least three repetitions, and as many as required to master technique as judged by the physiotherapist. This trial’s DB&C exercises consist of two sets of 10 slow-flow breaths to maximum inspiratory capacity with two to three inspiratory sniff breath stacking manoeuvres [61]. Each breath is held for 3 to 5 s. Each set of 10 breaths is followed by three coughs, or a forced expiratory technique with an open glottis called a ‘huff’, with a small firm pillow pressed over on the abdominal incision to support the wound and to encourage greater expiratory force. Participants will be encouraged to practice these exercises prior to their operation to develop familiarity.

### Standardisation of pre-operative interventions

The information booklet content will remain consistent between participating centres, although the formatting may change for site-specific requirements. All participating pre-operative physiotherapists will be required to view a scripted audio-visual recording of the pre-operative intervention prior to recruiting their first patient. They are instructed to adhere to the overall themes and premises of information delivery as included within the protocol script and video. Years of experience, seniority grade, and numbers of participants seen by each physiotherapist will be reported.

Ideally, interventions will be provided in person at PAC within six weeks of the scheduled surgery. However, in keeping with a pragmatic approach, if an eligible patient or physiotherapist is unable to attend PAC, patients can be enrolled, randomised, and provided with the interventions on another convenient day, or via telephone, prior to surgery. The mode of delivery will be recorded and the total proportion of telephone sessions will be reported. If a participant’s operation is delayed and the time from Pre-Op physiotherapy to day of surgery becomes greater than 42 days, a physiotherapist will contact the participant by phone for a review assessment and to remind them to read the booklet as provided at PAC. Participants allocated to the intervention group will, in addition, have a review of the education session and the DB&C exercises repeated over the phone.

### Standardisation of post-operative procedures

At the first available opportunity following surgery, all participants will be seen by a physiotherapist for a standardised assisted ambulation session (see Table 3). Ambulation is defined as marching on the spot beside the bed or walking away from the bedside for more than 1 min. Once a patient is ambulant for more than one minute, an Allied Health Assistant (AHA) will conduct all further ambulation sessions. If an AHA is unavailable, then a physiotherapist will continue to provide assisted ambulation. Health professionals (profession and years of experience) delivering ambulation will be reported. Participants will be seen once daily until discharged from physiotherapy services using defined scoring criteria [62] (see Table 4) or until discharged from hospital.

**Table 3:** LIPPSMAck POP ambulation protocol

Stage 1 (Safety)	Sit over edge of bed/sit in chair minimum of 2 min
Stage 2 (Safety)	March on spot 0–1 min
Stage 3 (Ambulation)	March on spot/walk away from bedside 1–3 min
Stage 4 (Ambulation)	March on spot/walk away from bedside 3–6 min
Stage 5 (Ambulation)	Walk away from bedside 6–10 min
Stage 6 (Ambulation)	Walk away from bedside 10–15 min
Stage 7 (Ambulation)	Walk away from bedside >15 min

**Table 4:** Discharge from physiotherapy scoring tool [62]

Mobility	Score
Reached pre-operative ambulation status	3
Requires supervision, status has plateaued	2
Requires assistance, status is improving	1
Unable to ambulate	0
Breath sounds	
Reached pre-operative levels and within expectations for that patient	3
Slightly decreased breath sounds or presence of a few added sounds	2
Markedly abnormal breath sounds and/or significant added sounds	1
Secretion clearance	
Able to clear secretions independently OR at pre-operative status	3
Requires assistance to clear secretions	1
SpO_2_% (on room air or pre-op oxygen levels)	
SpO_2_ ≥ 92 % (no respiratory condition) OR SpO_2_ ≥ 88 % (existing respiratory condition)	3
SpO_2_ < 92 % (no respiratory condition) OR SpO_2_ < 88 % (existing respiratory condition)	2
Respiratory rate (at rest and during activity)	
Within normal expectations	3
Outside acceptable range for the individual	2
Total score (min 6, max 15)	
A score ≥14 = discharge from physiotherapy	

At each session the participant will be progressed sequentially through the ambulation protocol stages aiming to achieve a walking time of more than 10 min at an intensity of at least three on the Borg 10-point visual analogue scale of perceived exertion [63] and where breathing is deeper than at rest. If necessary, ambulation sessions can comprise intervals at a work/rest ratio of 1:1. Shorter, but not longer, rest times are allowable. The final achieved ambulation stage is the total amount of time walked, not including rest periods. If participants are unavailable or unable to achieve ambulation for more than 1 min, the assisted ambulation session will be attempted again later in the day. Reasons will be recorded where participants are unable to ambulate or do not achieve a minimum of 10 min walking. Physiotherapists and AHAs will be provided with protocol prompt cards and trained by the site investigator.

At the first ambulation session participants will be provided with a walking aid if required, an abdominal support pillow for use during coughing, and a brief reminder to perform DB&C exercises as described within the information booklet provide pre-operatively. If a participant has forgotten his/her booklet, a new one will be provided. Participants will be encouraged to ambulate frequently to aid in the prevention of PPCs and encouraged to seek assistance from a nurse if necessary and to walk with their visitors. There will be no further provision of DB&C, PEP devices, incentive spirometers, or NIV by physiotherapists or AHAs.

Additional ambulation occasions outside physiotherapy assisted sessions or other ward staff encouraging patients to perform respiratory exercises will not be measured or controlled, as this would not be feasible. Both are considered standard ward care for both control and intervention group participants. However, if a participant is provided with an incentive spirometer or PEP device, this will be immediately removed. The break to protocol and duration of access to device will be recorded.

All other aspects of patient care, including pre-operative preparation, general anaesthesia, intraoperative ventilation parameters, fluid delivery, prophylactic antibiotic prescription, pain management, use of lines and drains, general nursing care, and discharge planning, will be provided at the discretion of nurses and physicians according to routine clinical practice at each participating centre.

### Blinding

Pre-admission clinic nurses and physiotherapists aware of group allocation will not have contact with participants post-operatively. A trial participation sticker (excluding group allocation) will be placed in the medical record. All post-operative ward staff, physiotherapists, PPC assessors, doctors, surgeons, nurses, discharge planners, data analysts, and statisticians will be blinded to group allocation. If a treatment group participant informs the assessor of their pre-operative education session, this will be noted and reported.

It is anticipated that patients will consider the pre-operative physiotherapy assessment and provision of a booklet an acceptable ‘sham’ treatment. This will be measured by interviewing a convenience sample of 30 consecutive participants via a semi-structured interview on their fifth post-operative day, or on the day of discharge, whichever comes first. They will be asked which group they believed they had been allocated to and, to test fidelity of the intervention over the control, what they remembered from their pre-operative physiotherapy session. The success of participant and therapist blinding will be tested and reported by requiring post-operative physiotherapists, AHAs, and assessors to guess group allocation for each of these 30 participants.

### Withdrawal from trial

Participants will be withdrawn for either of the following: (i) failure to progress to surgery within the first 3 months of PAC attendance or (ii) withdrawal of consent. All withdrawals and reasons will be reported.

### Primary outcome

The primary outcome is the development of a PPC within the first 14 post-operative hospital days. PPCs will be diagnosed with the Melbourne Group Scale (MGS) diagnostic scoring tool, which is reliable and valid following UAS and thoracic surgery [5, 8] and has high inter-rater reliability [64]. This tool has eight clinical criteria: four factors relating to symptoms and four to diagnostic markers (Table 5). A PPC will be diagnosed when four or more factors are present from midnight to midnight on one post-operative day.

**Table 5:** MGS PPC diagnostic criteria with modifications*

Diagnosis confirmed when four or more of the following are present:
Clinical factors
• New abnormal breath sounds on auscultation different to pre-operative assessment • Production of yellow or green sputum different to pre-operative assessment • Pulse oximetry oxygen saturation (SpO_2_) <90 % on room air on more than one consecutive post-operative day • Raised maximum oral temperature >38 °C more than one consecutive day
Diagnostic factors
• Chest radiograph report of collapse/consolidation. *When a CXR has been taken but no report is available, a ward medical officer or a senior respiratory physiotherapist with more than 10 years’ experience will be asked to report • An unexplained WCC greater than 11 × 10^9^/L • Presence of infection on sputum culture report • Physician’s diagnosis of *pneumonia, URTI, or an undefined chest infection, or prescription of an antibiotic for a respiratory infection

* modification made to original criteria

Participants will be assessed prospectively and daily for a PPC by a blinded assessor until the seventh post-operative day. Thereafter, additional PPC assessments are performed only as clinically suspected until day 14 when there are signs or symptoms of respiratory system deterioration reported within the medical record. To reduce the potential for missing data, retrospective collection of PPC data from the daily medical record will be permitted when a patient or assessor is unavailable for PPC assessment. The proportion of retrospective assessments will be reported. Components will be collected via the patient’s medical record and pathology/radiology databases. Diagnostic components (chest X-ray (CXR), white cell count (WCC), sputum microbiology) are recorded only if results are available. All medical officers are masked to group allocation and these diagnostic tests are ordered only as clinically indicated, and not routinely for the purposes of the LIPPSMAck POP trial.

For this trial, modifications (* in Table 5) have been made to diagnostic criteria to ensure that respiratory therapy will not be withheld longer than necessary from patients who may have developed a PPC. A CXR can be verbally reported by a blinded senior respiratory physiotherapist or ward physician, rather than awaiting a radiologist report. When three factors (out of a possible eight) in the MGS PPC tool are present, the blinded assessor or ward physiotherapist will contact the surgical ward doctor and discuss the option of further diagnostic testing to rule in or out a PPC. Additionally, these patients will be assessed twice daily to monitor clinical criteria for any deterioration.

A positive diagnosis of a PPC will be confirmed by a blinded senior physiotherapist, and the participant will then receive respiratory treatment as determined by the ward physiotherapist.

### Secondary trial outcomes

**Fig. 2:**
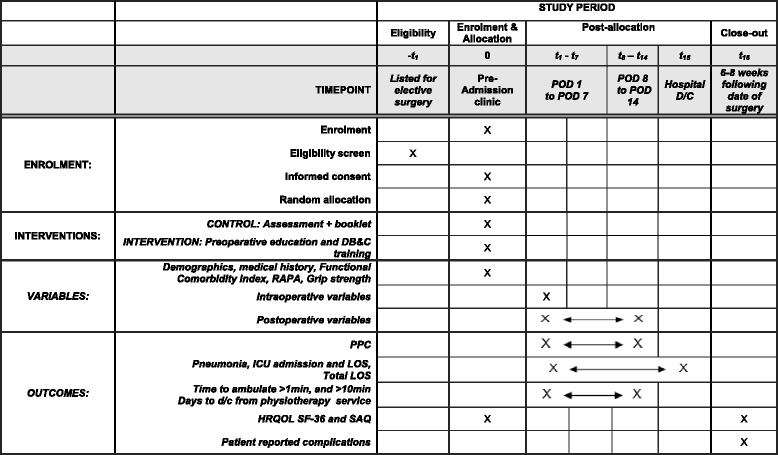
LIPPSMAck POP participant timeline and schedule of events. Describes LIPPSMAck POP participant timeline and schedule of procedures. Abbreviations: POD postoperative day, D/C discharge, DB&C deep breathing and coughing, RAPA Rapid Assessment of Physical Activity, PPC postoperative pulmonary complication, ICU intensive care unit, LOS length of stay, HRQOL health-related quality of life, SAQ Specific Activity Questionnaire

Secondary outcomes (Fig. 2) are:

1. Days of hospital length of stay (LOS). This is defined as the continuous time spent in any type of inpatient hospital service (acute care, sub-acute rehabilitation, and time at another hospital) from the day of admission to the day of discharge to a community dwelling
2. ICU LOS in days;
3. Unplanned ICU admission at any time point during the acute stay;
4. Pneumonia, defined as the presence of new CXR infiltrates along with at least two of the following criteria: temperature >38 °C, dyspnoea, cough and purulent sputum, altered respiratory auscultation, and WCC >14,000/ml or leukopenia <3000/ml [65] on any day within the first 14 post-operative hospital days;
5. Time in hours from end of operation to time able to achieve ambulation greater than 1 min;
6. Time in days from end of operation to post-operative day able to achieve ambulation greater than10 min;
7. Time in days to discharge from physiotherapy service (Table 4) [62];
8. Time in days to readiness for discharge from hospital as defined by standardised scoring criteria [66];
9. Hospital costs for the UAS admission episode of care. This will be supplied by the participating centres’ or health departments’ costing data for each participant’s admission episode.
10. Patient-reported complications at 6 to 8 weeks following day of surgery using a standardised semi-structured interview; and
11. HRQoL using the SF-36 and functional capacity using SAQ [58, 67] at 6 to 8 weeks following day of surgery.

Post-hospital discharge follow-up of self-reported complications, SF-36, and functional capacity will be via phone interview with a site investigator at 6 weeks from the date of surgery. If patients are unable to be contacted by phone for a period of five consecutive working days, a standardised cover letter, questionnaires, and self-addressed return paid envelope will be posted to the participant. Forms not returned within 2 weeks of posting will be considered lost to follow-up for the post-discharge secondary outcomes.

## Data collection

### Pre-operative variables

To measure baseline characteristics the following variables will be collected directly from the patient or the medical record: centre of recruitment, age, gender, height (cm), weight (kg), body mass index (kg/cm^2^), planned surgical procedure, category (hepatobiliary/upper gastrointestinal, colorectal, renal and urology, vascular, or other) and reason for the procedure, physical health status according to the American Society of Anaesthesiologists (ASA) and rated by the attending anaesthetist at the PAC (score 1 to 5), chemotherapy during the preceding 6 weeks, presence of a nasogastric tube before operation, respiratory status (auscultation signs and patient report of a daily productive cough), cough strength and presence of sputum (patient is asked to cough forcibly, the physiotherapist makes a subjective scoring of strength, effectiveness, and presence of sputum), sputum class (mucoid, mucopurulent, purulent) and colour using a validated colour chart tool [68] of any observed or patient reported regularly produced bronchial secretions, patient-reported history of a chest infection in the previous 14 days and if antibiotics had been prescribed, smoking history (non-smoker, current smoker, or ex-smoker having ceased more than 8 weeks pre-operatively), smoking pack years (1 pack year = 20 cigarettes per day for 1 year), years since smoking cessation, SpO_2_ (%) on room air, heart rate (beats per minute), co-morbidities as documented in the medical record (history of stroke or any other type of debilitating neurological disease, diabetes, arthritis, osteoporosis, asthma, COPD or other type of chronic respiratory disease, history of an acute myocardial infarct or angina, peripheral vascular disease, upper gastrointestinal disease such as reflux or gastric ulceration, current depressive illness or anxiety/panic disorder, visual or hearing impairment), patient’s self-report if the listed comorbidities significantly limit their walking on a day-to-day basis, Functional Comorbidity Index score [69], HRQoL with the SF-36, patient-reported estimated maximum metabolic equivalent (MET) physical activity using a self-rated physical Specific Activity Questionnaire (SAQ) [58], patient-reported measure of physical activity status using the Rapid Assessment of Physical Activity (RAPA) questionnaire and categorised to sedentary, under active, under active regular light activities, under active regular, and active [57], patient-reported maximum walking time along flat ground at comfortable walking pace, any limiting factor for mobilisation, and maximum grip strength as measured on the dominant hand using a calibrated hand dynamometer (Jamar Plus+; Sammons Preston, Rolyon, Bolingbrook, IL) performed with patients seated with shoulders adducted, elbows flexed to 90°, and forearms in the neutral position. The dynamometer handle position will be set to the second position for all tests [70], and three tests will be performed with verbal encouragement with the best test result recorded.

### Intra-operative variables

The following variables will be collected from the anaesthetic record, operation report, and medical record: duration of anaesthesia during surgery in minutes; mechanical ventilation parameters including mode of ventilation, level of pressure/volume control, and PEEP; average FiO_2_ during surgery; type and amount of intraoperative fluid delivered (ml/kg/h); numbers of blood transfusion units; prophylactic antibiotic delivery (medication and dosage); incision type (midline, unilateral subcostal, bilateral subcostal, transverse, combined thoracotomy, other). If there are multiple incisions used, the patient’s incision is categorised according to the closest abdominal incision to the thorax.

### Post-operative variables

Post-operative data will be collected daily for 14 days or until discharge from hospital, whichever occurs first: time in days from the pre-operative physiotherapy session to the operation; PPC risk stratification (low or high) using a defined risk calculation tool [5]; location (ICU, surgical ward, other) and duration in days at each location; days of analgesia and type (epidural, constant opioid infusion, patient controlled analgesia (PCA), patient controlled epidural analgesia (PCEA), oral, local pain infusion, or other); unplanned ICU/HDU admission and length of total ICU/HDU stay; length in days of total hospital stay; hours of mechanical ventilation; fluid delivery in the first 24 h (ml/kg/h); days and type of vasopressor use; hours and type of NIV use; days and types of oxygen therapy use; days, type, and indication for use for antibiotics; days and types of all drains and lines; day and diagnosis of a prolonged post-operative ileus using a standardised criteria [71] of 2 or more of the following factors in a 24-h period including nausea/vomiting, inability to tolerate normal diet, absence of flatus, abdominal distension, radiologic confirmation, and physician diagnosis of ileus.

Early ambulation parameters will be collected, including: time in hours from end of surgery until time to ambulation >1 min; post-operative day walked longer than 10 min; maximum rating of perceived exertion during ambulation at each session; maximum ambulation stage attained at each session (Table 2); number of assisted ambulation occasions; reasons for a patient being unable to participate in an ambulation session.

### Sample size

Sample size was calculated using inference for proportions comparing two independent samples with a 0.05 two-sided significance level and will have 80 % power to detect a 10 % absolute difference in PPC between Pre-Op (estimated at 10 %) and an education booklet (estimated at 20 %) when the sample size is 398. This is further increased by 11 % to account for attrition, resulting in a final sample size of 441.

### Data management

Data will be collected from participants using a standardised electronic case report form (CRF) and stored in participating centres’ password protected electronic hard drives. To ensure data quality the CRF has been designed with extensive use of data entry limitation rules and on-screen prompts to ensure correct data entry. Primary and secondary outcome data entry fields will be highlighted and required for completion of each participant’s data set. Automated weekly prompts will remind site investigators to complete any missing data points.

All site investigators will be trained directly by the principal investigator on correct administration of the trial. Site investigators will be required to perform random covert audits of data collected by trial personnel during the trial for reliability and correctness against the medical record. Once each participant’s data set is completed, it is de-identified, entered into a central database, and maintained securely by the principal investigator. All data, consent forms, and relevant correspondence will be stored according to Australian and New Zealand privacy laws and archived at trial sites for a minimum of 7 years. There are no industrial contractual arrangements in relation to the de-identified data. On completion of the trial, the database will be made available for independent analysis or as an appendix in the publishing journal if requested.

### Statistical methods

The prognostic strength and size of imbalances to potential confounding baseline variables between groups will be assessed. Adjustment covariates will be selected by backward stepwise regression from covariates that may have the potential for clinically significant alterations in effect sizes. These include: history of a respiratory comorbidity, smoking history, self-reported physical activity levels, age, BMI, length in time of operation, operation category (upper gastrointestinal, colorectal, urological, other), ICU admission immediately following the procedure, incision type and location [72], intraoperative ventilation strategies [4, 73], fluid delivery [74], blood transfusions [75], mode of post-operative analgesia [76], and use of prophylactic antibiotics [50].

All outcomes are to be analysed using intention-to-treat. The absolute and relative rates of PPC in the trial groups will be estimated using multivariate robust random effects Poisson generalised linear regression to allow assessment of binary outcomes with or without adjustment for potential confounding variables (incidence rates and rate ratios, 95 % confidence intervals, *P*-values). Treatment centre will be treated as a fixed variable in the multi-level models. In addition, the effect of time from the end of surgery/anaesthesia to commencement of symptoms of PPC will be compared using Cox proportional hazards regression with and without covariate adjustment (hazards ratio, 95 % confidence intervals, *P*-values). Graphic representation of this analysis will be performed using the Kaplan-Meier method.

Binomial secondary outcomes, including pneumonia, unplanned ICU admission, and patient reported complications, will be analysed using mixed effects Poisson regression. Secondary outcomes with irregular distributions, including length of time periods (ICU and total post-operative LOS, time to ambulation for 1 and 10 min, and time to discharge from assisted ambulation physiotherapy service), HRQoL, and functional capacity, will be evaluated for group differences using mixed effects ordered logistic regression, with mean time (95 % CI) estimated for descriptive purposes using mixed effects linear regression, with or without log transformation depending on distribution. Hospital costs associated with the interventions will be compared using mixed effects linear regression. Log transformation of highly skewed cost data will be performed.

An intention-to-protocol sensitivity analysis will be performed by excluding from the analysis any participant who did not undergo the anticipated scheduled upper abdominal surgery defined as a 5-cm incision or longer above, or extending above, the umbilicus. For example, this will include those participants who were scheduled for open surgery yet went on to only have a laparoscopic procedure or where the open incision remained wholly below the umbilicus.

The sensitivity of the outcome estimates to missing data will be evaluated using multiple imputation (Stata command syntax mi). All analyses will be performed using Stata version 13 or later (StataCorp, College Station, TX, USA).

### Data monitoring

The steering committee consists of the principal investigator and three academic supervisors who contribute to design and revision of the study protocol. The principal investigator is responsible for study administrative management and communication with local investigators, and for assisting participating centres with trial conduct, record keeping, and data management. An independent Data and Safety Monitoring Board (DSMB) consisting of a senior academic, staff anaesthetist, and biostatistician monitors the ethics of the study in accordance with the Declaration of Helsinki, overseeing safety and conduct of the study. This study compares two education-based treatment strategies that are highly unlikely to be related to serious adverse events (SAEs), though local investigators at participating centres remain responsible for reporting SAEs directly attributable to the intervention or control to the DSMB for review and consideration for referral to the institutional ethics review board.

### Duration and timeline

All 441 patients will be recruited by October 2015. Data collection will be completed, analysed, and the manuscript prepared for submission by March 2016. The final manuscript will be written in accordance with the CONSORT extensions for a pragmatic trial using a non-pharmacological intervention.

## Discussion

Studies in major UAS that have used the same PPC diagnostic tool as our group have reported a PPC rate of 13–18 % across all types of UAS [5, 6] with a specific rate of approximately 40 % in high-risk patients [8, 9]. Due to high incidence rates and costs of PPCs to patients and health care systems, there is great interest in their prevention.

Several clinical trials have compared a variety of different types and combinations of interventions to prevent PPCs. Trials demonstrating improvements in PPC rates have used multimodal interventions, so it is difficult to determine which component is effective in reducing PPCs, or indeed, if it is necessary to provide the whole ‘package of care’ to gain a significant benefit. This may influence resource provision, as providing the full package of therapy exactly as studied to gain the reported reduction on PPC rates may not be feasible, could be costly, and, indeed, may not be necessary in its entirety. Previous clinical trials have demonstrated that a single pre-operative education session can reduce PPC incidence to as low as 6 %, compared to a no-treatment control group rate of 27 %, *P* < 0.001 [47, 48], though assessors were un-blinded and potential confounders were not reported. Further, these trials were conducted 10–15 years ago, and changes in surgical and perioperative care have been significant in this time. The potential to significantly reduce the incidence of a high impact complication such as a post-operative respiratory complication with a low-cost and easily provided intervention of a single pre-operative physiotherapy session is appealing. It may not be ‘how much’ physiotherapy that is important, but rather ‘when’ that physiotherapy is provided. Unfortunately, conclusive evidence to support this hypothesis is lacking.

The LIPPSMAck POP trial is the first randomised controlled study powered and designed to investigate whether Pre-Op education and training reduces the incidence of PPCs. This RCT has been specifically designed to address previous methodological shortcomings in clinical trials investigating this intervention. Eligible participants are all patients listed for elective upper abdominal surgery and are representative of the heterogeneous nature of patients listed for these procedures. To ensure generalisability of results, the intervention will be delivered pragmatically and reflect current service delivery in Australia and New Zealand. Pre-operative education will be provided by a range of physiotherapists with different experience levels, including supervised students. The intervention and control has been designed and standardised to be provided by a physiotherapist of any experience level.

The active control of being assessed by a physiotherapist and receiving an identical subjective and objective interview and booklet, was chosen instead of a no-treatment comparator to specifically control for the Hawthorne effect. The LIPPSMAck POP trial standardises assisted early ambulation services and removes all physiotherapy coached respiratory therapy and provision of lung expansion devices post-operatively. We have not attempted to control for ambulation initiated by the participant or lung expansion exercises provided by nursing or medical staff and, in practice, this would be extremely difficult to achieve. However, with effective random allocation and blinding of post-operative staff members, it is reasonable to expect that patients in both the control and the intervention group will have an equal chance of having similar exposure to these factors. Regarding other known confounders such as pain management strategies, fluid administration, and intraoperative ventilation strategies, we have not attempted to standardise these due to the feasibility of doing so across three sites. Instead, the impact of potential perioperative confounders will be evaluated during statistical analysis and reported.

The primary outcome, PPC, will be measured by assessors masked to group allocation; all post-operative ward staff responsible for the delivery of all physiotherapy, medical, nursing, and general care and discharge planning will also be masked. The success of blinding procedures will be measured and reported. In modern health care delivery it is also important to consider the impact of an intervention on patient reported quality of life and not just on objective clinical outcomes [77]. It is hypothesised that if Pre-Op physiotherapy education is effective in reducing the incidence of a PPC, this may improve post-surgical recovery. Improvements in recovery may influence HRQoL following discharge from hospital, particularly physical functioning domains, as has been demonstrated previously [59]. LIPPSMAck POP will be measuring 6-week post-discharge patient reported complications, HRQoL, and functional capacity to estimate the potential effect that PPCs may have on these outcomes.

In conclusion, the LIPPSMAck POP trial is an investigator-initiated, bi-national, multi-centre, pragmatic, double-blinded, randomised controlled trial, powered and rigorously designed to test the hypothesis that pre-operative physiotherapy education prevents post-operative pulmonary complications in patients following major upper abdominal surgery.

### Trial status

The trial is ongoing and is actively enrolling.

## Abbreviations

AHA: Allied Health Assistant
ASA: American Society of Anaesthesiologists
COPD: chronic obstructive pulmonary disease
CRF: case report form
CXR: chest X-ray
DB&C: deep breathing and coughing
D/C: discharge
ERAS: Enhanced Recovery After Surgery
FiO_2_: fraction of inspired oxygen
HDU: high dependency unit
HRQoL: health-related quality of life
ICU: intensive care unit
LOS: length of stay
MET: metabolic equivalent
MGS: Melbourne Group Scale
NIV: non-invasive ventilation
PAC: Pre-Admission Clinic
PCA: patient controlled analgesia
PCEA: patient controlled epidural analgesia
PEP: positive expiratory pressure
PEEP: positive end-expiratory pressure
POD: post-operative day
PPC: post-operative pulmonary complication
RAPA: Rapid Assessment of Physical Activity
RPE: rating of perceived exertion
SAQ: Specific Activity Questionnaire
SF-36: 36-Item Short Form Health Survey
SpO_2_: pulse oximetry oxygen saturation
UAS: upper abdominal surgery
URTI: upper respiratory tract infection
WCC: white cell count
